# Using a Telegram chatbot as cost-effective software infrastructure for ambulatory assessment studies with iOS and Android devices

**DOI:** 10.3758/s13428-020-01475-4

**Published:** 2020-09-28

**Authors:** Michael Barthelmäs, Marcel Killinger, Johannes Keller

**Affiliations:** Universität Ulm, Abteilung Sozialpsychologie, 89081 Ulm, Germany

**Keywords:** Ambulatory assessment, Telegram, Chatbot, Open source, Python

## Abstract

In this work, we present an innovative and cost-effective approach to run ambulatory assessment (AA) studies on participants’ smartphones via Telegram Messenger. Our approach works both for Android and iOS devices. The population of potential participants in a given country or region consists of all individuals who (a) are in possession of a smartphone, (b) are willing to install Telegram Messenger, and (c) live in an environment providing constant connection to the Internet. In our new approach to AA, participants are asked to subscribe to a Telegram chatbot that provides them with links to brief surveys at specified points in time in their everyday lives via short notifications. We developed a user-friendly Python script that allows for the flexible editing of the chatbot’s settings, e.g., the number of surveys per day. All common survey software designed for mobile devices can be used to present surveys to participants. This means that data collection takes place exclusively via the selected survey software, not via Telegram. With our approach, AA studies can be carried out among iOS and Android users cost-effectively and reliably while data security is ensured. Initial data from a pilot study show that studies of this kind are feasible, and the procedure is accepted by participants. Our Python script is licensed under General Public License (GPLv3) and therefore freely available and editable: https://github.com/Raze97/Telegram-Survey-Bot

## Introduction

Ambulatory assessment (AA) studies can test psychological theories in everyday life. This offers the opportunity to evaluate the generalizability of theories and to examine boundary conditions that cannot be examined in the laboratory (cf. Fahrenberg, Myrtek, Pawlik, & Perrez, 2007; Trull & Ebner-Priemer, 2014). Hence, AA studies are one important tool to improve the understanding of human experience and behavior by capturing self-reports (e.g., Engeser & Baumann, 2016), observations (e.g., Ellis-Davies, Sakkalou, Fowler, Hilbrink, & Gattis, M., 2012), as well as motoric (e.g., Tryon, 2005) and physiological signals (e.g., van Lier et al., 2020) in the field. For about a decade, AA studies that focus on self-reports are typically implemented via participants’ smartphones (e.g., Killingsworth & Gilbert, 2010). Given the fact that more and more people possess a smartphone (cf. Miller, 2012), the feasibility of such studies should improve, however, carrying them out is still associated with a number of restrictions and pitfalls.

First, most of the available software solutions to conduct such studies are fee-based and therefore inaccessible to researchers or students with insufficient financial resources (see Table 1 for an exemplary overview of software solutions for AA studies). Second, the development and maintenance of self-made smartphone applications to run such studies can consume many resources, which means that this option is also not feasible for all researchers or students. Third, in order to collect non-selective samples, it would be ideal if all owners of a smartphone could participate in an AA study, regardless of the hardware and software (i.e., iOS or Android) they use. If participants use their own smartphone for participation (and no study smartphone is provided), there may be incompatibilities between the study software and participants' smartphones (cf. Trull & Ebner-Priemer, 2020), which limits the selection of possible participants. Fourth, participants might be concerned regarding the protection of their data and they could perceive the installation of an AA application on their private phone as an intrusion of their privacy, as applications can potentially collect a variety of information, like the number of incoming calls, GPS information, or total time of smartphone usage (cf. Miller, 2012).

**Table 1:** Exemplary overview of software solutions for Ambulatory Assessment-studies

Software	Operating system	Costs	Special features
PIEL Survey	Android & iOS	Free	Data are stored at the participants’ phones and must be sent to researcher at the end of the study
Lifedata	Android & iOS	Fee-based	Allows responses from one person to trigger questions sent to a “partner”
SurveySignal	Android & iOS	Fee-based	Survey links are provided via SMS
Experimetrics	Android & iOS	Fee-based	Basic version is free
mEMA	Android & iOS	Fee-based	Participants can upload images, videos, and audio files
Moviesens	Android	Fee-based	Option to collect physiological data
ESM Capture	iOS	Fee-based	-

*Note.* This overview does not claim to be comprehensive

In this paper, we present a costless approach that provides researchers with an option to collect ambulatory self-reports conveniently and reliably. Using this approach, participation is possible with iOS and Android devices, while data security for participants is ensured. We use the cloud-based instant messaging service Telegram as a platform to conduct AA studies. Specifically, we use a Telegram chatbot (i.e., software that conducts simple and automated conversations via textual methods) to provide participants with surveys in their everyday lives. We control the actions of this chatbot through a Python script, which allows researchers to set up study designs both flexibly and user-friendly. This script can be edited under Windows, macOS, and Linux, and can therefore be used by almost anyone. In the following paragraphs, we outline what requirements our approach has for participants, what demands are made on researchers, how our approach works in detail and how to set up a study with our approach.

## How our approach works for participants

To take part in a study with our AA approach, participants must meet the following requirements: (a) possession of a smartphone (with Android or iOS operating system), (b) Telegram Messenger app available or willingness to install it, and (c) availability of permanent mobile Internet access to receive messages from the chatbot and to open the online-survey links. If participants meet these requirements, they are instructed to add the study chatbot into their Telegram contact list. By subscribing to the chatbot, participants do not have to provide personal data, so neither the telephone number nor the Telegram username is stored. When the study begins, this chatbot sends messages to participants’ smartphones containing online-survey links.

## How our approach works for researchers

To run a study with our AA approach, researchers need (a) to create a Telegram chatbot account, (b) a software that can present online surveys at participants’ mobile devices, and (c) a computer/server that configures the actions of that chatbot through our Python script. This computer can use Windows, macOS, or Linux as our Python script works independently of the operating system. Further, this computer/server needs a constant Internet connection and must run continuously while a study is being conducted. We provide convenient download versions of our script. Importantly, our script is licensed under General Public License (GPLv3), which means that it is freely available and that it can be modified by others but must be freely available again after modification.

In the following sections, we explain the functioning of our AA approach in detail and describe the editable features of the chatbot. We further discuss how to set up an AA chatbot when Linux is used as the operating system for the server that controls the action of the chatbot. Please note that we do not elaborate on which software should be used to present online surveys.

## Components of our approach

Our Python script is the centerpiece of our approach and consists of three components: the configuration handler, the database handler, and the bot component itself. The script can be configured using a JSON^1^ file. In this configuration file, researchers can enter attributes like the survey links, the dates and times for scheduling the surveys, and the API^2^ token (see Fig. 3). The database is necessary, as a Telegram chatbot only sends messages in response to a user request. For our purpose, however, it is essential that the chatbot sends messages to chats at specific times without immediate user request. We therefore use a SQLite^3^ database, which allows storing the individual chat IDs of participants and to contact them through the Telegram bot API.

**Fig. 1:**
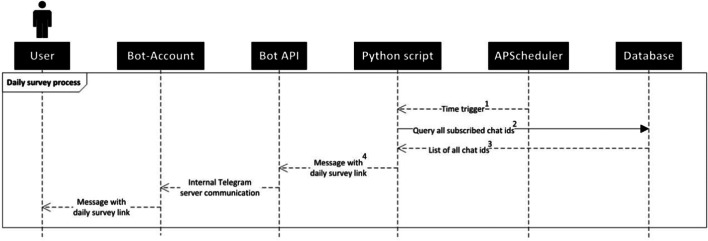
Sequence of actions providing participants with a daily survey link. Our script is accountable for steps (1) to (4), subsequent steps are executed by the bot API

We now describe the sequence of actions using the example of a daily survey, i.e., a survey link that is sent to participants multiple times throughout a study (see Fig. 1).

Let us assume that our participants are already subscribed to the chatbot, which means that their anonymous chat ID has already been stored in the database: (1) Based on the configuration file entries that can be edited by the user, the APScheduler package provokes time triggers. (2 + 3) The Python script then queries all subscribed chat IDs from the database. (4) Following this, the Python script sends a survey link to the bot API, which delivers the survey link to all subscribed chat IDs via the bot account.

## Available settings in our approach

Researchers can adjust various parameters in the configuration file to set up a study. As AA studies typically consist of a start survey, a daily survey, and one or more end surveys, the configuration file offers placeholders for the respective links of these surveys. Further, researchers can decide whether there is a fixed start date for all participants to begin with the study, or whether participants can start with the study flexibly within a given time period. Participants can also be randomized into different groups allowing to provide different groups with specified start, daily and end surveys, which enables experimental AA studies. Daily surveys can either be sent to participants at fixed times or depending on the time of getting up. The time lag between two survey links can be kept constant or fluctuate within a certain interval. Further, it is possible to provide participants with the option to request survey links on demand, which allows conducting event-based AA studies. Finally, researchers can send out multiple end surveys, hence, follow-ups can be scheduled flexibly.

## How to set up an AA chatbot with our script under Linux

**Fig. 2:**
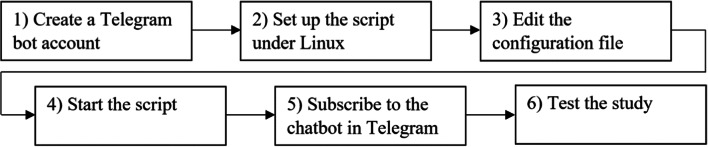
Sequence of actions to set up an Ambulatory Assessment chatbot under Linux.

In this paragraph, we describe the steps to set up an AA study when the server that controls the actions of the chatbot runs with Linux (even though we offer Windows and MacOS versions of the script, we recommend using the Linux version). Specifically, we describe the setup when using *Ubuntu Server* as Linux distribution and the terminal multiplexer *tmux*. The sequence of the setup procedure is depicted in Fig. 2**.**

**Fig. 3:**
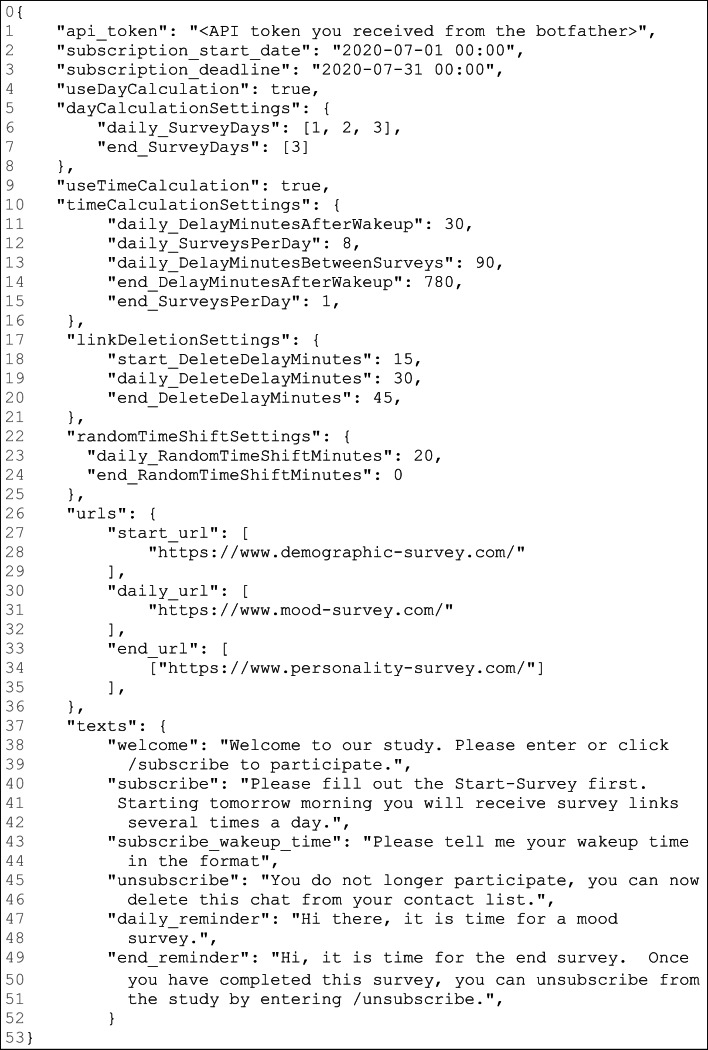
Excerpt from the configuration file, which allows controlling the actions of the chatbot. For demonstration purposes, we depict a reduced *config.json*-file here. Please note that our script only works with a complete file

1. Create a Telegram bot account: Open Telegram with a smartphone, web, or desktop application. A Telegram bot account can be created and configured with the so-called *BotFather*. To start a conversation with the BotFather, add the *@botfather* account in Telegram. A new bot is created entering the command */newbot*. You will then be asked to give the bot a name and a username. The name will be displayed in the contact list. The username is unique and ensures that the bot can be identified. After naming the bot, you receive the API token, which allows our Python script to communicate with the bot. To make using the bot easy, relevant commands to control the bot can be suggested. This is done with the command */setcommands* and can contain the following features: subscribe - subscribe to the survey; unsubscribe - unsubscribe from the survey; help - send me help; survey - send me a daily survey.
2. Set up the script under Linux: Download the latest release of our script from https://github.com/Raze97/Telegram-Survey-Bot/releases/. Unzip the file to a folder location of your choice.
3. Edit the configuration file: The file *config.json* is located in the directory *config* and allows to edit the settings for an AA study*.* Open the configuration file with the editor of your choice (e.g., nano) and adjust the settings according to your needs.
  Using a simple fictitious study, we demonstrate how this editing process works in principle. Figure 3 shows how a corresponding configuration file would have to look like. In the following description of the study, the numbers given in square brackets correspond to the code line that allows adjusting the respective feature in the configuration file (Fig. 3). It is important to note, however, that Fig. 3 does not show a complete but only a reduced configuration file for demonstration purposes. Please note that our script only works with a complete file. You can find the latest documentation of the complete configuration file under: https://github.com/Raze97/Telegram-Survey-Bot/wiki/Instruction-Linux#The-config-file.
  Let us assume that we want to set up the following study: Participants are allowed to start with the study between July 1, 2020 and July 31, 2020 [code line: 2, 3]. They are asked to fill in a start survey that queries demographic variables directly after participants subscribed the bot [cl: 28]. The message delivering the start survey is available for 15 min [cl: 18]. Participants’ mood is captured on three consecutive days, beginning one day after subscription [cl: 6], at eight times per day [cl: 12]. Participants receive messages with mood surveys depending on the time of getting up [cl: 9, 31]. They receive their first mood survey 30 min after they got up [cl: 11] and then, every 90 min [cl: 13]. The interval between two surveys is not fixed, but randomly varies between 80 and 100 min [cl: 23]. Each message containing a link to a daily mood survey is visible for 30 min [cl: 19], and afterwards it is deleted. At the evening of the last survey day [cl: 7], participants receive one end survey [cl: 15] 30 min after the last mood survey [cl: 14]. This end survey contains a personality questionnaire [cl: 34] and the message delivering it is visible for 45 min [cl: 20]. Study-specific texts help to make participation as pleasant and comprehensible as possible [cl: 37–51]. The API token received from Telegram must be copied into the corresponding field [cl: 1].

(4) Start the script: After editing the configuration file, switch back to the directory where the *config* folder is located. Make the Survey-Bot file executable with the command: *sudo chmod +x Survey-Bot.* To let the bot run in the background, the terminal multiplexer *tmux* can be used (please note, that under Linux there are also other terminal multiplexer options available). A new tmux-window can be opened with the command: *tmux*. Run the bot with the command: .*/Survey-Bot*. To detach the tmux-window use the key-combination: *ctrl + b, d*. In case you would like to stop the bot, use the key combination: *ctrl + c* and close the connection with: *logout.* To keep overview of the *tmux-*windows, assign a name to the window. To do this, the index number of the tmux-window must be queried with the following command: *tmux ls.* You can rename the tmux window with the command: *tmux rename-session –t <index number> <name>.* To check whether the bot is still running, enter the command: *tmux attach –t <index number or name>.*
(5) Subscribe to the chatbot in Telegram: Open the Telegram application and add the chatbot to your contact list. To do this, search for the unique username you have assigned to the chatbot. Press *Start* and enter the command */subscribe* to subscribe the chatbot.
(6) Test the study: We recommend a test run to check whether the chatbot really functions as intended. If the test run is positive, the actual study can be started. Researchers can remain subscribed to the chatbot even during the real study and can thus keep an eye on whether it is running as desired. If you want to unsubscribe to the bot, enter the command */unsubscribe* and delete the bot from your contact list.

## Pilot study

We conducted a small pilot study using our approach. The aim of this study was to get a first impression of whether our approach works as planned and how participants evaluate it. In terms of content, we examined the experience of flow in everyday life (cf. Csikszentmihalyi, 1975) on three consecutive days. Since the content of the study is not of relevance in the present context, we limit the report of the study results to respondents’ evaluation of our approach.

### Method

#### Participants

We tried to recruit as many participants as possible from a psychology course. This resulted in a sample of *N* = 15 (11 = women, 3 = men, 1 = other; M_age_ = 23.4, SD_age_ = 2.34). All participants started the study on the same day. All participants possessed a smartphone with constant connection to the Internet and were willing to install Telegram Messenger or were already using it. The sample consisted of iOS users (*N* = 4) and Android users (*N* = 11). All participants agreed to an informed consent and received a chocolate bar as compensation.

**Procedure**. Participants subscribed to the study chatbot during the course and filled out a start survey containing an instruction to create an individual participant code. The initial survey also contained several psychological scales and questions regarding demographic information. On the three following days, participants received eight survey links daily between 9 am and 9 pm. The interval between two survey links randomly fluctuated between 50 and 130 min (i.e., 90 min on average). The content of these daily surveys was identical for each measurement point. To ensure matching between different measurement points, participants’ code was queried at the beginning of each daily survey. At the evening of day three, participants received an end survey link, which gave them the possibility to evaluate our AA approach. Unipark survey software was used to create all online surveys.

#### Evaluation of our AA approach

We asked participants to evaluate our AA approach with open response format questions and with five closed questions. A sample item for a closed question is, “The surveys did not bother me in my everyday life”.

### Results

The compliance rate was satisfying, as participants replied to 87% of the daily surveys. In course of 3 days, 360 surveys were sent out to participants. These surveys were answered in 314 cases. There was no data loss due to technical problems.

#### Evaluation of our AA approach

Participants indicated that the survey did not bother them in their everyday lives. The descriptive statistics for the evaluation items are depicted in Table 2. Participants needed 83 s (SD = 50.77s) on average to answer a daily survey. The feedback on the open response format questions was consistently positive. While one participant for example said, that eight surveys a day were too many, two participants explicitly stated that eight surveys a day were fine.

**Table 2:** Evaluation of our Ambulatory Assessment approach

Question	Mean	SD	Median
(1) The surveys did not bother me in my everyday life.	3.77	0.73	4.00
(2) The surveys have kept me from important activities in my everyday life.	1.92	0.76	2.00
(3) It was hardly possible to complete all the surveys.	2.54	1.13	3.00
(4) I enjoyed participating in the study.	3.38	0.96	4.00
(5) I would not participate in such a study again.	2.38	1.26	2.00

*Note. N =* 13 (two participants did not fill in the end survey). Responses were given on a five-point Likert scale ranging from *not at all true (= 1)* to *completely true (= 5)*.

## Discussion

Running an AA study poses a challenge in many ways. We took efforts to circumvent some of the main challenges in the process to realize AA studies via smartphones by providing a costless Python script that allows researchers to collect ambulatory self-reports conveniently and reliably with a Telegram chatbot. The results of our pilot study suggest that participants generally accept our approach, when ambulatory self-reports are collected via Telegram. As the examined sample was rather small and therefore the results are essentially qualitative in nature, future studies should conduct further evaluations with larger samples using our approach.

Our approach is a flexible AA tool, as our script allows adjusting the following parameters: (a) placeholders for a start, daily and end survey link, (b) fixed or variable start date of the study, (c) randomization of participants to *n* different groups allowing to provide *n* groups with specified start, daily and end surveys (which enables experimental AA studies), (d) definition of fixed time-points for daily surveys, (e) possibility to use participants’ usual wake-up time to schedule daily surveys individually, (f) option for participants to request surveys on demand. Further, participation is possible with iOS and Android devices.

The requirements to use our AA approach are manageable both for participants and for researchers. Besides our Python script, researchers need (a) to create a chatbot account, (b) a software that can present online surveys on participants’ mobile devices, and (c) to set up a computer/server that configures the actions of that chatbot through our Python script. Although our approach is not a complete solution for AA studies (i.e., surveys must be prepared on a separate platform), its implementation is rather easy, as many researchers already have access to online-survey software (cf. Sassenberg & Ditrich, 2019). In addition, only limited resources are needed to create a chatbot account and to set up a computer/server as platform for our Python script as it can be edited under Windows, macOS, and Linux. To take part in a study using our approach, participants need (a) to own a smartphone, (b) to be willing to install Telegram Messenger, and (c) a constant connection to the Internet. As already described, the number of smartphone users and access to mobile Internet is constantly growing (cf. Miller, 2012), which will make recruitment of large sections of the population even easier.

It is important to emphasize that we do not want to advertise Telegram. Our only goal is to provide a reliable infrastructure for AA studies using participants’ smartphones. Telegram seems to be an appropriate means to realize AA studies for the following reasons: (a) Participants are provided with software updates for the messenger application which renders our approach compatible with different hardware and operating systems even in the long run, (b) users have the option to keep their personal data largely undisclosed (i.e., users can deny Telegram access to their contacts), (c) compared to *WhatsApp*, the implementation of chatbots is possible and supported, and (d) the implementation effort for our approach was minimal, since we only had to program the back end. That is, we only programmed the functions that run in the background of the software. It was not necessary to develop the front end, like a graphical user interface (i.e., an app) or to manage notifications. Additionally, we did not have to go through the effort of publishing an app.

One decisive advantage of our approach lies in the separation of the reminder infrastructure (Telegram) and the survey infrastructure. That is, participants get a reminder to fill in a survey via Telegram, but they do answer the surveys on a different platform. Accordingly, no survey information is passed on to Telegram. Furthermore, researchers using our approach are never in the position to access other contents of participants’ mobile devices. Moreover, it is not possible for participants to communicate with other participants via the chatbot.

Comparing our approach to already-existing tools, the main advantages are the user-friendly and cost-free use, the data security level, and the fact that both Android and iOS users can participate. To the best of our knowledge, PIEL survey is the only feasible cost-free AA tool currently on the market. While the PIEL survey has the advantage that data can also be collected if the participant does not have access to the Internet, this presupposes that participants independently send their data to the researcher at the end of the study, which comes with the risk of a complete data loss. Furthermore, participants’ personal data and their answers to the survey are less sharply separated than it is the case in our approach, as the PIEL survey application is installed on participants’ smartphones.

Another important feature of our approach is that it is licensed under GPLv3. This means that third parties have free access to our code and can modify it as needed, but they commit themselves to make their code freely available again. This way we have laid the foundation that our approach can be further developed and improved (and at the same time remains freely available), a process to which we enthusiastically invite interested colleagues. A possible development of our approach could include, for example, that surveys can be carried out directly in Telegram and not via additional survey software whereby, of course, data protection for the participants must be guaranteed.

### Conclusions

In sum, we think we have taken an important step forward by rendering the essential research method of Ambulatory Assessment via smartphones available to many researchers and students who previously had no chance of conducting such studies.

## Footnotes

1 JavaScript Object Notation: A lightweight data-interchange format.

2 Application Programming Interface: An interface between different parts of a computer program. Specifically, the API token allows our Python script to communicate with one specific Telegram chatbot account.

3 SQLite: A relational database management system contained in a C library.
